# Two Attitudes towards Textuality in International Law: The Battle for Dualism

**DOI:** 10.1093/ojls/gqac010

**Published:** 2022-06-11

**Authors:** Jean d’Aspremont

**Keywords:** international law, international legal theory, interpretation, critical theory, post-structuralism

## Abstract

This article sketches out two distinct attitudes towards textuality in international law, namely international hermeneutics and international poetics. It argues that these two attitudes towards textuality espouse very different types of dualism of thought. This difference bears major implications on how the international lawyer approaches international legal texts. In exposing these two attitudes towards textuality and the distinct types of dualism they reveal, this article makes a plea for a greater embrace of international poetics by international lawyers, and thus for a complete remoulding of international lawyers’ dualist patterns of thought.

## 1. Introduction

This article sketches out two distinct attitudes towards textuality in international law. One of them is actual whilst the other is aspirational. One is at a dead end and yet casually perpetuated by international lawyers. The other is the only hope that international lawyers—and especially international legal scholars—have left to take the textuality of which they are in charge seriously. The actual and ill-fated attitude towards textuality is called here *international hermeneutics*. The aspirational and hopeful attitude towards textuality is labelled *international poetics*.

Notwithstanding their dramatic differences, international hermeneutics and international poetics have in common a resort to dualist patterns of thoughts. In fact, as it is argued here, both of them are manifestations of the strong dualism that has informed international lawyers’ engagements with textuality since the advent of modern international law. And yet, as this article will show, albeit articulated around dualist patterns of thoughts, these two attitudes towards textuality espouse a very different type of dualism. In exposing these two attitudes towards textuality and the distinct types of dualism they reveal, this article makes a plea for a greater embrace of international poetics by international lawyers, and thus for a complete remoulding of international lawyers’ dualist patterns of thought.

The article is structured as follows. The second section considers the omnipresence of dualist patterns of thought in international legal thought, which the two attitudes towards textuality examined here are manifestations of. The third section describes the actual hermeneutic attitude towards textuality, namely international hermeneutics, and elucidates the type of dualism it is built on. Section 4 introduces the aspirational attitude towards textuality, namely international poetics, and sheds light on the dualism around which it is articulated. The last section makes a plea for a greater embrace of international poetics and the dualist patterns of thought it puts in place, simultaneously showing that this may not be as revolutionary as it sounds, for the type of dualism at the heart of international poetics may already be at work in international legal thought and practice.

## 2. Prolegomena: Dualism of Thought

Postulating binaries and articulating one’s claims in dualist terms, like this article does, is a common pattern of thought. In the West, dualism of thought is usually traced back to Descartes, who allegedly put forward the first articulated form of dualism.^1^ Indeed, Descartes is remembered for his distinction between the thing-in-itself that is accessible to the self and knowable by the self on the one hand and all the representations of the thing-in-itself that are necessarily relative and contingent on the other.^2^ Yet, Cartesian dualism is only one of the many variants of dualism of thought. Kant, for his part, rejecting the possibility of an absolute knowing by the self of the thing-in-itself but still acknowledging the possibility of a thing-in-itself, put forward a type of dualism less dogmatic and more transcendental which turns the attention to the relation between the thing-in-itself and its representation.^3^ It is well known that Hegel came to repudiate the dualisms of both Descartes and Kant by seeking to show that dualism is bound to be caught in idealism as only the idea provides the self with access to the thing-in-itself, idealism simultaneously enabling the possibility of absolute knowledge.^4^ After Hegel, dualism of thought continued to thrive, no less than through Marxism,^5^ and later through structuralism,^6^ the latter possibly being dualism of thought at its best. It can be said that, in the West, the history of modern thinking until today can be read as a history of dualist thinking.^7^

For sure, this article, by postulating two attitudes towards textuality in international law, belongs to and continues this long philosophical dualist tradition. It must be made clear that such ancestry is nothing sensational, for the entirety of the international legal literature, at least in the English language, is built on dualist patterns of thought that bear some important kinship with the philosophical tradition that has just been described here. In fact, it seems possible to claim that dualism of thought has shaped and ordered^8^ international legal thought since its modern inception in the 18th century.^9^ This is a finding that unsurprisingly^10^ continues to apply to contemporary doctrinal and orthodox works on international law. It could equally be extended to all those recent critical works that shed light on the contradictions and false necessities of international legal argumentation, as well as those compelling studies of the ideological, neo-colonising and masculine dimensions of the world-making performances of international law.^11^

The two attitudes towards textuality that are examined in the following sections, namely international hermeneutics and international poetics, are similarly exhibiting a strong kinship with dualism. For reasons that are discussed in the next section, it is probably international hermeneutics that epitomises most explicitly the use of dualist patterns of thoughts.^12^ Yet, it will be shown below that international poetics, although resolutely seeking to disrupt the type of dualism that informs international hermeneutics, remains caught in dualist patterns of thoughts too.^13^ In that sense, even if this article’s promotion of international poetics seeks to gainsay a dominant type of dualism that is found in international legal thought and practice, it does not aim at vindicating a breakaway from the dualist tradition in international law, not least because it defines international poetics in opposition to international hermeneutics. In that regard, this article is premised on the idea that there may be no way out of dualist thinking,^14^ which is why the international lawyer must constantly seek to renew her dualist patterns of thought.

It must be preliminarily stressed that the exposition by this article of two distinct attitudes towards textuality as well as of the two types of dualism that inform them is not entirely novel. Literary theory and 20th-century philosophy have long distinguished between two attitudes towards textuality. For instance, Barthes claimed that there are two ways in which one can engage with a piece of literary work: one that seeks to map and catalogue all its distinct meanings and one that seeks to pick just one meaning.^15^ In the same vein, Deleuze, speaking about the act of writing about texts, distinguished between those who territorialise themselves within the territory of a state of things which they deem established and those who write to become alien to writing.^16^ Derrida similarly drew a distinction between two types of interpretations of texts: one that dreams of deciphering a truth and that experiences the necessity of interpretation as a banishment and another that is informed by an attempt to go beyond man and humanism.^17^ Yet, whilst the two attitudes towards textuality discussed here, and their dualist patterns of thought, have long been distinguished in literary theory and philosophy, they have found little echo in international legal thought. It is the ambition of this article to elucidate the implications of such distinction in relation to international law texts with a view to promoting a new attitude towards textuality in international law, one that helps renew the latter’s dualist tradition.

A final terminological and definitional remark is in order. The distinction between hermeneutics and poetics is itself borrowed from critical literary theory.^18^ It obviously draws on concepts that have a long history and accordingly does not do justice to the many attitudes towards textuality that could possibly be referred by either hermeneutics or poetics. In that sense, the distinction between hermeneutics and poetics inevitably simplifies the great variety of interpretive practices at work in law and beyond. Yet, this article is premised on the idea that the distinction between hermeneutics and poetics constitutes a formidable tool to shed light on dramatically diverging attitudes towards international law texts.

## 3. The Actual Attitude towards Textuality: International Hermeneutics

This section describes an attitude towards textuality that is deemed dominant in international legal thought and practice and that is called here international hermeneutics (section 3A).^19^ It then elaborates on the type of dualism that such attitude towards textuality denotes and which is called here *linear dualism* (section 3B).

### A. International Hermeneutics and the Delivery of a Pre-existing Meaning

From the perspective of international hermeneutics—as is broadly understood here, any word (eg ‘immunity’, ‘reparation’, ‘torture’), idiom (eg ‘armed attack’, ‘*opinio juris*’, ‘*jus cogens*’, ‘*force majeure*’), aphorism (‘the parties aim to reach global peaking of greenhouse gas emissions as soon as possible’, ‘every internationally wrongful act of a State entails the international responsibility of that State’, etc) or text (article 53 of the Vienna Convention on the Law of Treaties, article 38 of the Statute of the International Court of Justice, etc) of international law always stands for a thing, an idea, a norm, a practice, a behaviour, an institution, a discourse, etc. To put it differently, for international hermeneutics, the words, idioms, aphorisms and texts of international law—that is, what is called here the forms of the international legal discourse^20^—perform a signifying function whereby they represent a thing, an idea, a norm, a practice, a behaviour, an institution, a discourse, etc. As a result, in international hermeneutics, those words, idioms, aphorisms and texts are approached as carrying and delivering meaning, a meaning that must constantly be searched for. For international hermeneutics, the forms of the international legal discourse^21^*mean*, thereby necessitating that the international lawyer constantly seeks to elucidate and extract what such forms mean.

It should not be surprising that international hermeneutics, as it is understood here, dramatically bears upon the way in which international lawyers construe and practise interpretation. Indeed, from the perspective of international hermeneutics, interpretation boils down to an activity centred on a quest for meaning.^22^ In other words, for international hermeneutics, interpretation is always reduced to meaning-determination: it is an activity geared towards the determination of the meaning carried and delivered by the forms of the international legal discourse, which includes the determination of the meaning of their contexts, their origin, their authors, their addressees, their effects, etc. In international hermeneutics, the interpreter’s main task is thus to question the text, that is, to interrogate the text as to which of the meanings available in the interpreter’s repertoires^23^ of meaning it carries.^24^

It must be acknowledged, once more, that the understanding of interpretation from the perspective of international hermeneutics, as it has been sketched out here, is not uniform, but comes in various shades of grey. One could, in some broad-brush strokes, distinguish between a strong and a soft variant of international hermeneutics as is understood here. The strong version of international hermeneutics posits that the meaning loaded onto the forms of the international legal discourse is never found at the surface of the form and ready to be delivered, but requires a careful process of extraction, the latter being commonly referred to as ‘interpretation’. According to this strong version of international hermeneutics, interpretation refers to the process of extraction of the pre-existing meaning that has allegedly been loaded onto the forms of the international legal discourse and which the latter are supposed to carry and deliver.

Even if this strong version of international hermeneutics may not necessarily correspond to an actual belief and often amounts to a casual and inarticulate disciplinary narrative, it has been extensively debunked in international legal literature for several decades, thereby giving way to what is called here a soft variant of international hermeneutics. Indeed, according to this soft variant, the extraction of the meaning that is loaded onto international legal forms is reduced to a theatrical performance,^25^ such performance being what constitutes the meaning of such forms.^26^ Likewise, the soft variant of hermeneutics calls for a greater emphasis on the reader as well as on the readership community,^27^ thereby further distancing itself from the strong version of international hermeneutics.^28^

Yet, even if the strong version of international hermeneutics has probably lost its thrust, the presupposition that the forms of the international legal discourse deliver meaning on which international hermeneutics is predicated has endured in the soft variant of international hermeneutics that populates international legal thought and practice. Indeed, notwithstanding the—nowadays rather mainstream—claims about indeterminacy of international law texts,^29^ about the performative effects of interpretation and about the primacy of the reader, the very postulation that the forms of the international legal discourse deliver meaning continues to be upheld across the board, for the form is still held as delivering meaning at one point or another. In that sense, international legal thought and practice, notwithstanding the common postulation that meaning is created in the process of extraction, continues to be dominated by a soft version of international hermeneutics by virtue of which the forms of the international legal discourse supposedly deliver meaning. Even the most critical takes on interpretation continue to be predicated, in one way or another, on such soft international hermeneutics and the idea that the forms of the international legal discourse simply mean.

### B. International Hermeneutics’ Linear Dualism

It is submitted here that international hermeneutics, as it has been sketched out above, is deeply engrained in the dualism that has shaped international legal thought for the last centuries. Indeed, international hermeneutics rests on a fundamental dualist engagement with international law by virtue of it distinguishing forms and meaning. In international hermeneutics, the signifying function bestowed upon the words, idioms, aphorisms and texts of international law, and thus all the forms of the international legal discourse, is only possible to the extent that such forms are distinct from the meaning they refer to, that is, from the thing, the idea, the norm, the practice, the behaviour, the institution, the discourse, etc they represent. In fact, meaning can only be represented, carried and delivered by the forms of the international legal discourse if it is strictly distinct from such forms. Said differently, it is only as long as the forms of the international legal discourse and the pre-existing meaning they carry and deliver are distinguished that it is possible to excavate an alleged pre-existing meaning and thus to carry out an interpretation of the forms of the international legal discourse. In that sense, from the perspective of international hermeneutics, the distinction between meaning and form is a condition of the international legal discourse.^30^ For that reason, it could be said that, in international hermeneutics, the dualism that accompanies the distinction between form and meaning is of an *existential* character.

The dualism that comes with the distinction between form and meaning at the heart of international hermeneutics is also strongly *hierarchical*, for it gives primacy to the meaning over the form. The dualism around which international hermeneutics is constructed not only entails that the forms of the international legal discourse are distinct from (and external to) the meaning they carry and deliver; it also makes the forms of the international legal discourse secondary thereto^31^ and derived therefrom.^32^ In fact, according to the dualism of international hermeneutics, there would be no forms in the international legal discourse if there were no pre-existing meaning to carry and deliver and from which forms could be derived. This is why pre-existing meaning comes to be construed, according to international hermeneutics, as the cause and origin of the forms of the international legal discourse.^33^

Lastly, the dualism that informs international hermeneutics thanks to its articulation around the distinction between form and meaning can similarly be said to be *differential*. In fact, in international hermeneutics as is understood here, the pre-existing meaning carried and delivered by the forms of the international legal discourse is supposedly retrieved through a system of differences between forms themselves.^34^ It is by virtue of the differences between forms that the specific meaning carried and delivered by a form of international law can be determined. For instance, ‘custom’ means what it means because, as a form, it is distinct from ‘treaty’; ‘armed attack’ represents what it represents because, as a form, it is different from ‘use of force’; ‘compensation’ for the sake of responsibility entails what it entails because, as a form, it cannot be conflated with ‘satisfaction’, etc. From this dominant perspective, the differences between international law’s words, idioms, aphorisms and texts constitute what allows the allocation of a pre-existing meaning to each and every form of the international legal discourse.^35^

The type of dualism at the heart of international hermeneutics, as it has just been described, brings about a very specific type of attitude towards international law texts. It is submitted here that, taken together, the existential, hierarchical and differential dimensions of the dualism informing international hermeneutics entail a linear type of reading of international law texts.^36^ Indeed, by virtue of such existential, hierarchical and differential dualism, international law texts are supposed to have a beginning—that is, the original meaning they carry, which is their cause and origin—and an end—that is, the ultimate meaning they carry and deliver. Meaning being both the beginning and the end of international law texts, reading international law texts thus requires a venture into the causes and origins of such texts that is followed by the experience of the delivery of their ultimate meaning. In other words, from the perspective of international hermeneutics, it is only by yielding to the beginning and the end of international law texts and proceeding in that order that the reader of the international law texts will come to capture the ultimate meaning of such texts. In that sense, it can be claimed that international hermeneutics’ dualism puts in place a mode of reading that turns international law texts into systems of lines.^37^

The specific mode of reading that is enabled by international hermeneutics could be illustrated as follows. Should an expert of international law and security be solicited to determine whether article 51 of the United Nations Charter (which is known for providing for ‘an inherent right of self-defence’ under international law) could be invoked to justify the use of force against non-state actors (eg insurgents), she will, following the attitude prescribed by international hermeneutics, start by distinguishing the text of article 51 and its content and presupposing that an original meaning was agreed upon and then inscribed in the text of article 51. Seeking to unearth that original meaning as well as the way in which this meaning has possibly evolved independently from the text of article 51 itself, the international lawyer will look at the way in which article 51 is possibly linked with other provisions of the Charter (eg article 2.4) from which the meaning article 51 could have possibly been derived. Still looking to elucidate article 51’s original meaning, the international lawyer will also look at the preparatory works of the Charter, possibly even taking the geopolitical context of the drafting of that document as a type of context that can help her determine the original meaning of article 51. Having so traced the origin and the cause of article 51, namely the original meaning that was allegedly inscribed in the text of that provision, the international lawyer will then look at the way in which this original meaning has possibly evolved since its inscription into article 51. This will entail mapping out the other meanings that have subsequently been inscribed in that text. To do so, she will, for instance, turn to the case law of some of the most authoritative judicial bodies that came to apply that provision, as well as the most authoritative scholarly commentaries of the provision. The mapping of these other and subsequent meanings will allow the international lawyer embracing the linear dualism prescribed by international hermeneutics to draw an overall semantic genealogy, one that began with the original meaning loaded into the text of article 51, deemed to be the cause and the origin of that provision, and that ends with the latest other meaning inscribed into that text. Whatever she eventually concludes as to whether article 51 applies to non-state actors, the international lawyer embracing international hermeneutics would thus have been distinguishing the meaning and the text before going on to draw lines between the former and the latter, between the original meaning and other subsequent meanings, and between these other subsequent meanings and the text of article 51.

## 4. The Aspirational Attitude towards Textuality: International Poetics

The previous section sketched out and illustrated what is deemed the dominant attitude towards textuality in international legal thought and practice, namely international hermeneutics, as well as the type of dualism of thought that permeates it. This section describes an alternative approach to textuality and a new type of dualism of thought, which is called international poetics. The section first defines international poetics as an attitude towards textuality based on the deferral of meaning by the forms of the international legal discourse (section 4A). It then sheds light on the distinct type of dualism around which international poetics is articulated, namely *spatial dualism* (section 4B).

### A. International Poetics and the Deferral of Meaning

International poetics is premised on the idea that the forms of the international legal discourse do not carry and deliver any pre-existing meaning and that meaning cannot be the cause and origin of such forms. From the perspective of international poetics, the words, idioms, aphorisms and texts of international law—that is, the forms of the international legal discourse—do not carry and deliver any pre-existing meaning but, instead, constantly postpone meaning, thereby condemning meaning to be permanently absent from forms. When asked to signify a thing, an idea, a norm, a practice, a behaviour, an institution, a discourse, etc, the forms of the international legal discourse constantly pass on the job of signification to other forms. When these other forms to which signification is passed are, in turn, asked to signify, they will similarly point away to yet other forms. In other words, when asked to signify a thing, an idea, a norm, a practice, a behaviour, an institution, a discourse, etc, the forms of the international legal discourse permanently *defer meaning* to other forms without such deferral process ever being completed and meaning ever being pinned down.^38^ Being perpetually deferred, meaning is eternally absent and nowhere to be found in the forms of the international legal discourse of which it cannot be the cause or the origin.^39^ By perpetually deferring meaning and ensuring its absence, the forms of the international legal discourse can thus no longer be secondary to meaning.^40^

Although the foregoing might run counter to the international lawyer’s most mundane attitude towards textuality, it must be stressed that the claim that forms postpone meaning by perpetually deferring the latter and thereby leaving the process of signification eternally unachieved is no novel affirmation and has long been advocated in literary theory and philosophy.^41^ Whilst it would be of no avail to summarise such literature here, there is one lesson from literary theory which is worth foregrounding for the sake of this section’s sketch of international poetics. In fact, literary theory and philosophy can teach the international lawyer that the above-mentioned permanent deferral of meaning and thus its perpetual absence in the international legal discourse entails neither the emptiness of forms nor a conflation between forms. Indeed, it is possible for the forms of the international legal discourse to have an identity of their own short of any ingrained or assigned pre-existing meaning. According to such argument, the forms of the international legal discourse have a meaningless identity, that is, an identity that cannot be reduced to any fixed or inherent meaning. The identity of forms short of their respective meanings is what has been called in literary theory and philosophy the forms’ *self-difference*.^42^ This possibility of upholding the identity of forms short of meaning is critical for the argument made here, as it demonstrates that moving away from international hermeneutics and embracing international poetics is not exclusive of each and every form of the international legal discourse having a distinct identity.

To appreciate how each and every form of the international legal discourse has an identity of its own despite it carrying and delivering no pre-existing meaning, two observations are warranted about what the identity of forms cannot possibly be. First, it should be repeated that the distinct identity of each and every form of the international legal discourse is not any kind of inherent pre-existing meaning or meaning in disguise. Second, and more fundamentally, it must be emphasised that the distinct identity of the forms of the international legal discourse is not simply and mechanically constituted by the relationships of difference with other forms, for this would, once again, reintroduce a presupposition of a pre-existing meaning.^43^

If not by virtue of any pre-existing meaning, of some meaning in disguise or of relationships of difference with other forms, how can a form be distinct from another form? In other words, what is the self-difference of forms if not some kind of pre-existing meaning, some meaning in disguise or the result of a system of differences? It is argued here that the difference with other forms is not *outside* the form concerned—that is, in its relationship of difference with other forms—but *within* each and every form. More specifically, according to such argument, each and every form differentiates itself from others by virtue of the otherness *within itself*. To take but a few examples from the international legal discourse, it is by virtue of such self-difference that ‘invalidity’ is not ‘wrongfulness’, ‘general principles of law’ are not ‘customary law’, an ‘armed attack’ is not a ‘use of force’, an ‘injured state’ is not a ‘non-injured state’, ‘responsibility’ is not ‘liability’, ‘*jus cogens*’ is not ‘*erga omnes’*, etc. Inhabited by what it is not, each and every form of the international legal discourse has an identity of its own.

The difference within the selfsame that informs the work of self-difference is sometimes captured through the notion of *trace*, which is said to ‘haunt’ the form.^44^ The trace is ‘the event of the other-in-the-law’.^45^ It indicates the vanishing presence of other forms of which it is a vestige.^46^ The trace has already disappeared when it is noticed. It is thus absent too.^47^ In that sense, the trace is a ghost of the other.^48^ The trace is itself porous, always unfinished and only traceable to other traces.^49^ It can accordingly never be encountered as an object or as data,^50^ let alone serve as a foundation^51^ or context^52^ of the form and of its self-difference. In other words, the trace is no surrogate for any pre-existing meaning, for it is always being induced and constructed in the process of deferral of meaning.^53^

Such a notion of trace—which expresses the work of self-difference, that is, the difference within the selfsame—is helpful to understand how self-difference allows the forms of the international legal discourse to have a distinct identity short of any pre-existing meaning or of a system of differences. Indeed, each form carries *the trace* of what it is not, that is, the mark of other forms that it is different from. It is the trace of other forms (which is a difference within the selfsame) that confers the identity to the form. As a result of this trace of what it is not, each and every form of the international legal discourse can be said to have a *divided identity*, such form being always already inhabited by other forms.

That the identity of forms is a divided identity by virtue of the trace of other forms calls for yet another important remark. Such divided identity cannot be a binary identity and the trace of the other cannot be the trace of an opposite.^54^ For sure, the forms of the international legal discourse are inhabited by the traces of other forms that seem at variance with—and that differ from—the form being inhabited. Yet, such difference ought not to be construed as an opposite, for doing so would reduce self-difference to meaning. Indeed, as long as the other that inhabits the form constitutes its opposite, there is a presupposition of a fixed meaning by virtue of which the opposition is constructed and apprehended. Said differently, construing self-difference as a binary identity, and thus understanding the trace of the other as a trace of the opposite, presupposes a pre-existing meaning, which is a move that empties international poetics as is understood here. From the perspective of international poetics, the other within the selfsame is not the opposite other but only *an* other.^55^ To give but one example, if the traces inhabiting the form ‘armed attack’ in the discourse on the use of force, be they ‘use of force’, ‘territorial integrity’, ‘peace’, etc, were to be understood as an opposite, that would amount to presupposing a pre-existing meaning to the form ‘armed attack’, one that would then contradict a pre-existing meaning vested onto ‘use of force’, ‘territorial integrity’, ‘peace’, etc.

It must be emphasised that international poetics does not entail that the search for the meaning of forms is simply supplanted by a search for the meaning of forms’ self-difference. Indeed, as was already indicated above, the forms’ self-difference does not constitute yet another content that can possibly be extracted, interpreted or created. Such traces of otherness within the selfsame are no surrogate for meaning. Actually, the moment traces of the other forms come within purview, they are already caught in the deferral of meaning and have vanished. In that sense, the trace of otherness within the selfsame, that is, self-difference, can never be apprehended, produced, given a content and interpreted. That does not entail that the forms of the international legal discourse are empty. On the contrary, the forms of the international legal discourse are always in a state of saturation because of the constant deferral of meaning: forms’ self-difference ensures that forms are always saturated with traces of those other forms to which meaning is deferred.

Here, too, the consequences of the attitude towards textuality that has just been outlined are best illustrated by reference to the way in which interpretation can be understood and practised from the perspective of international poetics. In fact, as can probably be anticipated, international poetics bears major consequences on the understanding and practice of interpretation. If the forms of the international legal discourse, as is posited by international poetics, neither carry nor deliver meaning, there are simply no meanings to be determined or found, be that of forms themselves or of their origin, context, authors, agendas, effects, etc. Said differently, from the perspective of international hermeneutics, interpretation cannot be about determining any meaning whatsoever, for the latter is always absent.^56^ This is why international poetics strips interpretation of its hermeneutic dimension,^57^ for any extraction, let alone imputation, of meaning is impossible.^58^ For the same reason, as far as international poetics is concerned, the identity of forms never raises a question of determinacy or indeterminacy. Indeed, a form never has a determinate or indeterminate identity, but only bears the—always vanishing—traces of other forms within it.

### B. International Poetics’ Spatial Dualism

It is submitted here that international poetics, although it does not repudiate dualism altogether, seriously qualifies the linear dualism of international hermeneutics. In fact, as is shown here, international poetics rests on a different type of dualism that is called spatial dualism.

International poetics’ rupture with international hermeneutics’ dualism can be summarised as follows. International poetics departs from international hermeneutics’ dualism in that it no longer elevates the distinction between form and meaning into a condition of the international legal discourse. Indeed, for international poetics, meaning is absent from the international legal discourse, which is entirely dominated by (and articulated around) forms referring to other forms in their deferral of meaning. Likewise, international poetics does away with international hermeneutics’ hierarchical dualism in the sense that it foregrounds the forms, and only the forms, unmaking the primacy of meaning over forms. Finally, international poetics repudiates international hermeneutics’ differential dualism in that the identity of the forms of the international legal discourse no longer lies in a system of differences of meanings between forms but in forms’ self-difference. In undoing the existential, hierarchical and differential dualism of international hermeneutics, international poetics breaks away from any type of linear dualism and ceases to reduce international law texts to systems of lines with a beginning and an end.

Such repudiation by international poetics of the linear dualism of international hermeneutics bears dramatic consequences on the attitude of the international lawyer towards international law texts. In fact, once the linear dualism of international hermeneutics is resisted, international law texts can be approached as spaces rather than lines. Free from the idea that the meaning pre-exists the form and is carried and delivered by it, international poetics particularly enables the international lawyer to appreciate that international law texts are *spaces* with no centre^59^ where something is happening.^60^ More precisely, if international law texts are no longer experienced as lines with a beginning and an end, they can be construed as sites of infinite passage^61^ or wandering spaces^62^ with thousands of entries and exits.^63^

Turning international law texts into spaces rather than lines is no minor rupture from the dualism of international hermeneutics. Indeed, such spatial understanding of textuality concretely entails a recognition of the multidimensionality of texts^64^ and a greater appreciation of the texts’ plenitude,^65^ as well as that of its infinite possibilities in terms of deferral of meaning.^66^ In doing so, international poetics frees the international lawyer from her obsession for the semantic secret of the text^67^ and ensures that the outcome of one’s reading is never predetermined.^68^ In that regard, it could be said that international poetics encourages a slow and meticulous reading^69^ that refuses any pre-assigned sender and pre-assigned recipient.^70^ Said differently, international poetics’ understanding of texts as spaces encourages a metonymical reading of such texts.^71^ Doing away with linear dualism, international poetics thus leads to an infinite and always-unfinished^72^ reading of international law texts.^73^

Importantly, however, approaching international law texts in a non-linear fashion does not amount to a complete repudiation of dualist patterns of thoughts. In fact, dualism remains very present in international poetics for a number of reasons. First, international poetics, just like international hermeneutics, still brings about a process of interrogation of international law texts, albeit not for single answers but for an appreciation of such texts’ plenitudes. This interrogative posture always carries a form of dualism, one that distinguishes between the interrogating reader and the interrogated text.^74^ Second, international poetics, despite flattening the distinction between forms and meaning as it condemns the latter to a permanent absence, upholds the distinctiveness of forms and does so by opposition to meaning. In other words, international poetics continues to be, just like international hermeneutics, articulated around a distinction between forms and meaning, albeit in a non-existential, non-hierarchical and non-differential way. For these reasons, it can be argued that, far from doing away with dualism, international poetics only gives volume to it^75^ by turning the lines of reading prescribed by international hermeneutics’ linear dualism into spaces of reading. This is why the type of dualism around which international poetics is articulated is called here spatial dualism.

An observation is in order regarding the type of reading of international law texts that is enabled by the spatial dualism of international poetics: the spatial dualism that permeates international poetics cannot lead to a reading that is *oppositional*. Such an observation is important because many international legal scholars who have come close to espousing an approach to textuality that corresponds to international poetics have simultaneously promoted an attitude towards textuality based on *oppositional reading*.^76^ Yet, as was indicated above,^77^ the self-difference of the forms of the international legal discourse—that is, what provides the identity to such forms—never amount to a binary identity. As was highlighted already, the trace of the other in the forms of the international legal discourse is never the trace of an opposite: the other within the selfsame is not the opposite other, but only *an* other. Construing self-difference as a binary identity, and thus understanding the trace of the other as a trace of the opposite, would presuppose a pre-existing opposite, and thus bring back the linear dualism of international hermeneutics.^78^ For that reason, the type of reading of international law texts facilitated by the spatial dualism of international poetics cannot be of an oppositional nature.

The work of the spatial dualism that accompanies international poetics, as it has been sketched out here, can also be illustrated by reference to article 51 of the United Nations Charter (which is famous for providing for an inherent right of self-defence). The international lawyer who is solicited to determine whether article 51 is applicable to non-state actors and who espouses the spatial dualism of international poetics will first refrain from presupposing that an original content had first been agreed upon and subsequently inscribed in article 51. She will still isolate the text from its content, but will not consider such a content to be preceding the text or to be the cause thereof. Likewise, she will come to ascribe no beginning and no end to article 51, let alone a semantic genealogy that begins with an original meaning loaded onto the text of that provision and ends with another meaning deemed more authoritative and subsequently inscribed into that text. Instead, she will construe the text of article 51 as a signifying space where content has always been and will continue to be eternally created by all those that have ever invoked article 51. As a result, the international lawyer sensitive to the spatial dualism of international poetics will see herself not as extracting or deriving a meaning from article 51, but as navigating an immense semantic space inhabited by the incalculable traces of other texts that themselves carry the incalculable traces of further texts. In doing so, she will realise that article 51 bears the traces of other texts that are both anterior and posterior to it, be it some preparatory works of the United Nations Charter or subsequent juridical or scholarly pronouncements about article 51, themselves referring to a myriad of other anterior or posterior texts, words, forms, etc outside the legal realm. Although conscious of the infinity of the semantic universe enabled by article 51 and of the absence of origin and end to anything that such provision could mean, the international lawyer amenable to international poetics will appreciate that only a slow and meticulous reading of the infinite possibilities of article 51 will simultaneously realise that the little sense she can make of the text of article 51 remains dependent on her own repertoires of available semantic possibilities, of the other texts that her reading of article 51 brings her to and even of her affects, her desires, her emotions, her loyalties, etc.

It is important to highlight that, in the above-mentioned example, being caught in the deferral of meaning at work in the signifying space of the text of article 51 of the United Nations Charter does not entail that the international lawyer sensible to international poetics will be unable to answer the question whether article 51 applies to non-state actors, let alone that she will be caught in a forever reading of that text and find herself unable to answer any pressing question about what that provision dictates. On the contrary, she will be able to make a better choice, not as to which fixed meaning is allegedly attached to the text of article 51, but as to which deferral of meaning ought to be entailed by article 51—and thus which other forms article 51 ought to be referring to, a choice that will more consciously mobilise her individual ethics. The maximisation of the ethical responsibility of the international lawyer when espousing international poetics is a point to which the final section of this article returns.

## 5. The Battle for Dualism: A Case for International Poetics

The previous sections have sketched out two attitudes towards textuality in international legal thought and practice, one that has been deemed dominant and called international hermeneutics and one that has been perceived as aspirational and called international poetics. In this last section, this article explicitly makes a plea for a greater embrace of international poetics and of the spatial dualism that accompanies it.

It is argued here that the international lawyer would gain much from letting international poetics guide her attitude towards textuality. The point made here is that international poetics, thanks to the spatial dualism it puts in place, allows the international lawyer to be true to her texts. Indeed, rather than perpetuating a theatrical quest for an ever-absent meaning, the international lawyer that embraces international poetics enables texts to be texts, namely spaces where something is always happening.^79^ It could even be said that the more the pre-existing meaning of the text is resisted by the international lawyer that espouses international poetics, the more volume is given to the text, for resistance to pre-existing meaning consolidates the text as a space of signification.^80^ Said differently, by shedding the presupposition that texts carry and deliver a pre-existing meaning, the international lawyer sympathetic to international poetics allows texts to be constantly reinvented and rejuvenated. The text, from the perspective of international poetics, is never frozen but permanently solicited by (and teeming with) the hopes for meaning of the international lawyer. In that sense, international poetics and its specific dualist patterns of thought make international law texts eternally new and constantly renewable.

That international law texts, from the perspective of international poetics, are eternally new and constantly renewable is rather good news for those pursuing something like reform.^81^ It must be acknowledged, however, that this may not be good news for everyone. In particular, the international lawyer trained in the hermeneutic tradition may not feel entirely comfortable with texts that are constantly reinvented. In fact, the international lawyer may want to live in a world where her texts are fixed with meaning once and for all, that is, a world where international law texts carry and deliver a predictable meaning that the international lawyer can mobilise to manage this very world.^82^ In that sense, international poetics can be experienced as a disempowerment of the international lawyer as well as a threat to the modern project of international law, including to the peace-guaranteeing, welfare-enhancing, individuals-protecting goals traditionally assigned to international law.

It must be conceded that international poetics and its spatial dualism make the international lawyer a permanent nomad in a textual universe.^83^ Indeed, international poetics invites the international lawyer to wander the spaces made of international law texts without a clear direction. Yet, it is of the utmost importance to highlight that the international lawyer turned into a nomad in the immense textual space of international law texts by virtue of international poetics finds herself neither in exile nor in a prison. Nomadism is the very condition that liberates the international lawyer from the linear dualism of international hermeneutics. In fact, the nomadism enabled by international poetics and its spatial dualism make the international lawyer appreciate how international law texts do what they do through the traces of other texts. At the same time, international poetics and its dualist patterns of thought make the nomadic international lawyer appreciate her ethical responsibility for what international law texts, as spaces, do to the world. Because deferral of meaning in the spaces of international law texts hinges on the forms available to the reader of the international law text, the international lawyer cannot just hide behind the words, idioms, aphorisms and texts of international law. International law texts do what they do—and they often do terrible things—by virtue of the deferral of meaning enabled by the international lawyer and, more specifically, the forms that the latter vest in the forms being interpreted. Being responsible for the deferral of meaning by the forms of the international legal discourse and for vesting forms in forms as she ventures into the space of international law texts, the international lawyer sympathetic to international poetics is inevitably instrumental in what the words, idioms, aphorisms and texts of international law do.^84^ In that sense, international poetics, far from entailing a withdrawal of the international lawyer from textuality and her abdication from any responsibility vis-à-vis the world,^85^ maximises her ethical responsibility, for the international lawyer comes to realise the role she plays in defining the type of deferral of meaning prompted by the text concerned and the consequences possibly attached to it.^86^

It could also be objected that international poetics and its spatial dualism, by leading the international lawyer to an infinite and always-unfinished reading of international law texts, contradicts the core functions that law, the lawyers and their texts are called upon to perform.^87^ It should be made clear here that international poetics, albeit leading to a slow, meticulous and metonymising reading, ought not to prevent the international lawyer espousing such an attitude towards textuality from taking a position and defending it. To put it differently, international poetics is not synonymous with utter relativism or immobilism. International poetics neither bars the international lawyer from being engaged nor precludes her from performing the possible functions expected of her. The difference with international hermeneutics rather lies with the fact that the international lawyer embracing international poetics would not defend a fixed meaning supposedly attached to the text, but rather a specific way in which the text defers meaning to other texts. In that sense, international poetics simply makes her aware that what she ought to defend is not a meaning that is always absent from the text, but a specific way in which the text refers to other texts, that is, a specific way to navigate the space created by the text for the sake of signifying.

One could similarly take issue with the failure of international poetics to capture and appreciate the processes of material inscription and communication of international law texts^88^ and its playing down of the importance of history and contexts.^89^ In the same vein, one could also rail against international poetics for making international law texts out of the world and for denying that such texts are worldly creations.^90^ At a time where the contingency of international law,^91^ its histories^92^ and its world-constituting performances^93^ are drawing much scholarly attention, international poetics could thus be deemed a very conservative and purely textualist^94^ endeavour. This objection, however, must be strongly rebutted. International poetics, by turning international law texts into spaces replete with the traces of other texts, captures the world, the processes of inscriptions of international law texts as well as the contexts of the making and reading of those texts much better than if those texts were just the containers of pre-existing meaning. Indeed, from the perspective of international poetics, the world, the processes of inscriptions of international law texts as well as the contexts of the making and reading of those texts are all already in international law texts whose space they inhabit. In other words, the world, processes of inscriptions of international law texts as well as their contexts inevitably inform anything than can be solicited from the text by the international lawyer venturing into the space of international law texts.

International poetics could also be perceived as condemning the international lawyer that embraces the spatial dualism thereof to some vile pragmatism and a resignation not to seek truth from the text.^95^ This objection must similarly be strongly refuted. In fact, construing the texts of international law as spaces, as international poetics posits, does not entail that anything goes.^96^ In international poetics, meaning cannot be deferred at whim and according to the preferences of the international lawyer precisely because interpretation and all that is vested in it—preferences, agendas, imagination, etc—are themselves caught in the deferral of meaning. The deferral of meaning always hinges on the other forms to which meaning is deferred at the moment the international lawyer solicits meaning from the form. In other words, in international poetics, the deferral of meaning is thus dependent on the other forms that are available to the international lawyer and that she sees traced in the text. This latter point is important, for it indicates that the approach to textuality that follows international poetics cannot be reduced to a replacement of the problems of hermeneutics by the pleasure of infinite creation^97^ as well as a glorification of the aleatory.^98^ The reading of international law texts enabled by international poetics is thus never aleatory, anarchical and accidental. It takes place in a wider universe that is itself saturated with signs that defer meaning to other signs.

A final argument can be made in favour of international poetics. It is an argument of a descriptive nature that turns international poetics into a naturalistic necessity.^99^ This argument goes as follows. One may question the extent to which international hermeneutics and its linear dualism have ever allowed the location and extraction of a pre-existing meaning in international law texts. Indeed, it may be that international hermeneutics has itself always been an aspiration for the international lawyer, one that has never succeeded in ensuring the location and extraction of meaning from international law texts. Said differently, it cannot be excluded that international hermeneutics has always been, at best, a ritual around international law texts. The continuous debates about interpretation in international law and the recurrent disagreement on whether the rules on interpretation actually constrain the extraction of meaning from international law texts may just be an illustration of the failure of international hermeneutics. Accordingly, international hermeneutics ought not to be discontinued, for it never was an actuality. At the same time, it may be that international poetics and its dualist modes of thinking, rather than being an aspirational attitude towards textuality, are already at work in international legal thought and practice. The international lawyer has possibly always approached international law texts as spaces with no centre, no beginning and no end. In that sense, international poetics has always been in action in international legal thought and practice. If this is the case, the descriptive claim about the state of the international lawyer’s current attitude towards textuality on which this article was premised and with which it started was all wrong. What was presented as the actual has always been the aspirational and what was presented as the aspirational has always been the actual. In other words, it may be that, in the midst of the battle for dualism narrated in the article, the international lawyer has never ceased to practise international poetics.

## Footnotes

1 It is interesting to note that, in pre-modern times, dualism of thought, and especially the understanding of Christ as having two distinct natures, while supported by Nestorianist school, came to be severely repressed by the Catholic Church after the Council of Chalcedon (451 AC) in favour of a monophysitist approach to the nature of Christ, whereby the latter would have only one inseparable nature, partly divine, partly human). For some remarks, see Roland Minnerath, *Histoires des Conciles* (Presses Universitaires de France 1996) 17–23. See also the remarks of Régis Debray, *Vie et mort de l’image* (Gallimard 1992) 99–141.

2 This is not to say that dualism of thought is unknown from pre-modern thought. Previously, the distinction between the body and the spirit brought about an important mode of dualistic thinking. See Jacques Le Goff, ‘L’homme médiéval’ in Jacques Le Goff (ed), *L’Homme médiéval* (Seuil 1989) 15.

3 Quentin Meillassoux has claimed that Descartes’s dualism is a type of dogmatic dualism, whereas Kant’s dualism constitutes a form of transcendental dualism. See Quentin Meillassoux, *Après la Finitude. Essai sur la nécessité de la contingence* (Seuil 2006) 13–49.

4 For an impressive and yet accessible interpretation of Hegel, see Jean Hyppolite, *Logique et existence* (Presses universitaires de France 1952).

5 On the dualism of Marxism, see Peter Singer, *Marx, A Very Short Introduction* (Oxford UP 2000) 27–30. See also the remarks of Régis Debray, *Le Scribe* (Editions Grasset et Fasquelle 1980) 161–70; Cornelius Castoriadis, *L’Imaginaire comme tel* (Hermann Editeurs 2007) 38.

6 On the dualism of structuralism, see Jacques Derrida, *L’écriture et la différence* (Seuil 1967) 426. See also the remarks of Jonathan Culler, *Structuralist Poetics. Structuralism, Linguistics, and the Study of Literature* (Routledge 2002) 5–6.

7 On the idea that modernity generalised modes of thinking articulated around identity and differences, see Timothy Mitchell, *Questions of Modernity* (University of Minnesota Press 2000) 17; Michel de Certeau, *L’écriture de l’histoire* (Editions Gallimard 1975) 59; Michel Foucault, *Les mots et les choses* (Gallimard 1966) 68.

8 On the idea of distinctions as powerful modes of ordering, see Gilles Deleuze and Claire Parnet, *Dialogues* (Champs essais 1996) 29; Roland Barthes, *Leçon* (Seuil 1978) 12; Bruno Latour, *Nous n’avons jamais été modernes. Essai d’anthropologie symétrique* (La Découverte 1997) 68–9.

9 On the extent to which international legal thought is articulated between constraints of justice on the one hand and constraints of concreteness on the other, see Martti Koskenniemi, *From Apology to Utopia. The Structure of International Legal Argument* (CUP 2005). On the extent to which international legal thought is based on dualistic self-referentiality, see Jean d’Aspremont, *International Law as a Belief System* (CUP 2017). See also Jean d’Aspremont, ‘Three International Lawyers in a Hall of Mirrors’ (2019) 32 LJIL 367.

10 On the idea that distinctions order their own contestation, see Derrida, *L’écriture et la différence* (n 6) 47; Foucault (n 7) 12; Latour (n 8) 56–7; Roland Barthes, *Mythologies* (Seuil 1957) 248. On the inevitable situatedness of critique, see Edward Said, *The World, the Text, and the Critic* (Harvard UP 1983) 26.

11 It is argued here that the specific resorting to binary oppositions and the use of oppositional reading witnessed in these critical works have perpetuated the centrality of dualist patterns of thoughts. On the idea that such critiques have relied on a certain type of dualism, be it the concreteness and the normativity, the centre and the periphery, the form and the content, the male and the female, the universal and the particular, etc, see the remarks of Rose Parfitt, *The Process of International Legal Reproduction. Inequality, Historiography, Resistance* (CUP 2019) 21; Ntina Tzouvala, *Capitalism as Civilisation. A History of International Law* (CUP 2020) 38–40; Zarbiyev (n 11) 109–18. See also the remarks of Akbar Rasulov, who argues that Martti Koskenniemi engagement with structuralism in *From Apology to Utopia* is much more on the side of traditional French(-speaking) structuralism and that it is more a structuralism *à la* Saussure. See Akbar Rasulov, ‘From Apology to Utopia and the Inner Life of International Law’ (2016) 29 LJIL 641.

12 See s 3B below.

13 See s 4B below.

14 See generally Jacques Derrida, *Positions* (Editions de Minuit 1972) 21, 35, and 56. See also Derrida, *L’écriture et la différence* (n 6) 46. On the dualist foundation of any science and philosophy of science, see Gaston Bachelard, *Le nouvel esprit scientifique* (PUF 1934).

15 Roland Barthes, *Critique et vérité* (Seuil 1999) 60.

16 Deleuze and Parnet (n 8) 89.

17 Derrida, *L’écriture et la différence* (n 6) 427.

18 On the distinction between a tradition based on hermeneutics and one based on poetics, see Culler, *Structuralist Poetics* (n 6) vii–viii. It is interesting to note that Edward Morgan (who similarly questions the very notions of meaning) speaks of aesthetics rather than poetics. See Edward Morgan, *The Aesthetics of International Law* (University of Toronto Press 2007).

19 This is what I have also called elsewhere ‘deliverability thinking’, which I deem to be a type of meaning-centrism. See Jean d’Aspremont, *After Meaning. The Sovereignty of International Legal Forms* (Edward Elgar 2021). On the idea that law is not only a form of inscription but also a system of delivery, see Peter Goodrich, ‘Europe in America: Grammatology, Legal Studies, and the Politics of Transmission’ (2001) 101 Colum L Rev 2033, 2066.

20 It should be acknowledged that forms should not be reduced to textual inscriptions. As far as legal forms are concerned, images, symbols, gestures, paintings, ceremonies, rituals, stained glass windows, etc are also forms which defer meaning. Forms are also in the oral language. The argument made here is limited to textual inscriptions. On non-textual inscriptions, see Jacques Derrida, *Papier Machine* (Galilée 2001) 384. On the idea that language, even oral language, is already a type of writing, see Catherine Malabou and Jacques Derrida, *La Contre-Allée* (La Quinzaine Littéraire 1999) 73–5. See also the remarks of Goodrich (n 19) esp 2069–84.

21 Discourse is understood here in a rather generic way as to refer to any argumentative practice about international law, be it judicial, academic, diplomatic or militant. On the notion of discourse, see generally Hayden White, *Tropics of Discourse: Essays in Cultural Criticism* (Johns Hopkins UP 1978) 4–5.

22 On the idea that interpretation is traditionally deployed as ‘technology of analytical knowledge’, see Pierre Legrand, ‘Siting Foreign Law: How Derrida Can Help’ (2011) 21 Duke J Comp & Int’l L 595, 598.

23 On the idea of interpretation as an action of inventorying, see Maurice Merleau-Ponty, *Signes* (Gallimard 1960) 260.

24 On the idea that concepts are deployed as identity checks, see TW Adorno, *Negative Dialectics* (Continuum 2007) 5. See also Henri Bergson, *La pensée et le mouvant* (Flammarion 2014) 229 and 240. On the idea of identity check in the scientific discourse, see Bachelard (n 14) 16. On the idea that questioning entails a dualist mode of thinking, see Deleuze and Parnet (n 8) 21 and 27.

25 On the metaphor of the theatre, see Stephen Humphreys, *Theatre of the Rule of Law: Transnational Legal Intervention in Theory and Practice* (CUP 2010).

26 See eg Ingo Venzke, *How Interpretation Makes International Law: On Semantic Change and Normative Twists* (OUP 2012).

27 For a classic, see Stanley Fish, *Is There a Text in This Class? The Authority of Interpretive Communities* (Harvard UP 1980). see also S Fish, ‘Fish v Fiss’ (1984) 36 Stan L Rev 1325.

28 See A Bianchi, ‘The Game of Interpretation in International Law: The Players, the Cards, and Why the Game is Worth the Candle’ in A Bianchi, D Peat and M Windsor (eds), *Interpretation in International Law* (OUP 2015); A Bianchi, ‘Textual Interpretation and (International) Law Reading: The Myth of (in)Determinacy and the Genealogy of Meaning’ in Pieter HF Bekker, Rudolf Dolzer and Michael Waibel (eds), *Making Transnational Law Work in the Global Economy—Essays in Honour of Detlev Vagts* (CUP 2010) 35. This used to be my position too. See Jean d’Aspremont, *Epistemic Forces in International Law* (Edward Elgar 2016); see also Jean d’Aspremont, ‘The Multidimensional Process of Interpretation: Content-Determination and Law-Ascertainment Distinguished’, in A. Bianchi, D. Peat and M. Windsor (eds.), *Interpretation in International Law* (OUP, 2015) 111–29.

29 For an overview of the state of the discourse on the indeterminacy of international law as well as some critical observations, see CA Miles, ‘Indeterminacy’ in Jean d’Aspremont and Sahib Singh (eds), *Concepts for International Law. Contributions to Disciplinary Thought* (Edward Elgar 2019) 447–58.

30 It is no coincidence that distinguishing the forms of the international legal discourse from the meaning they refer to and represent is probably one of the greatest achievements of modern legal thought as it consolidated itself in the 19th and 20th centuries. On the modern modes of representation, see generally Mitchell (n 7) 17–18. See generally Foucault (n 7) 58 and 70–2; Jacques Derrida, *De la Grammatologie* (Editions de Minuit 1967) 50; Latour (n 8) 24; Emmanuel Levinas, *Altérité et transcendence* (Fata Morgana 1995) 17.

31 On legal discourses’ common presumption of inconsequentiality and weightlessness of forms in the process of transmission of meaning, see Pierre Schlag, ‘“Le Hors de Texte, C’est Moi”. The Politics of Form and the Domestication of Deconstruction’ (1990) 11 Cardozo L Rev 1631, 1633.

32 On the idea of derivation of writing in Western thought, see Malabou and Derrida (n 20) 44–5.

33 On the traditional idea that the form is treated as weightless and inconsequential and that what matters is the substantive meaning, see Schlag (n 31) 1633. See also Vincent Forray and Sébastien Pimont, *Décrire le droit…et le transformer. Essai sur la décriture du droit* (Dalloz 2017) 104.

34 On the idea of system of differences, see Derrida, *L’écriture et la différence* (n 6) 426. See also the remarks of Culler, *Structuralist Poetics* (n 6) 5–6.

35 The determination of meaning through the differences between forms has been theorised in traditional structuralist linguistics, which has shown the extent to which the identity of the sign is relational and differential and can only be determined through differences with other signs. See generally F de Saussure, *Course in General Linguistics* (Charles Bally and Albert Sechehaye ed, Open Court 1986). On this aspect of the work of Saussure, see the remarks of Derrida, *L’écriture et la différence* (n 6) 427.

36 On linearity of thinking, see Derrida, *De la Grammatologie* (n 30) 122. It must be acknowledged that the linear dualism of international hermeneutics in international law has been challenged, albeit not to an extent that has questioned either dualism itself or international hermeneutics. See eg Anthony Carty, *The Philosophy of International Law* (Edinburgh UP 2007); Koskenniemi, *From Apology to Utopia* (n 9).

37 Compare with the arborescent modes of thinking in Deleuze and Parnet (n 8) 33.

38 In the same vein, see Goodrich (n 19) 2059. Compare with Edward Morgan’s questioning of the very idea of meaning. See Morgan (n 18) 4.

39 In the same vein, see Zarbiyev (n 11) 37–45. See also Goodrich (n 19) 2062. Edward Morgan also claims that legal doctrines have no beginning and exist in a hall of mirrors. See Morgan (n 18) 6.

40 Compare with the idea that signs work ‘despotically’ of Richard Harland, *Superstructuralism. The Philosophy of Structuralism and Post-Structuralism* (Routledge 1987) 124.

41 Derrida, *De la Grammatologie* (n 30) 11–126; Jacques Derrida, *Marges de la Philosophie* (Editions de Minuit 1972) 1–29; Derrida, *L’écriture et la différence* (n 6) 411. On this aspect of the work of Derrida, see the remarks of Peter Salmon, *An Event Perhaps* (Verso 2020) 12. See also Geoffrey Bennington, *Jacques Derrida* (Seuil 1991) 56. Compare with Roland Barthes, *Le bruissement de la langue. Essais critiques IV* (Seuil 1984); Roland Barthes, *S/Z* (Seuil 1970) 9–11. For an earlier, albeit less radical, contestation of the idea that language is the instrument of a pre-existing thought, see Edward Sapir, *Language. An Introduction to the Study of Speech* (Ishi Press 2014) 14–17.

42 Self-difference’ is one of the ways in which Derrida’s idea of *différance* has been translated in English. Simon Glendinning, *Derrida. A Very Short Introduction* (OUP 2011) 62. Compare with the translation of différance as *spacing* by Jonathan Culler, *On Deconstruction. Theory and Criticism after Structuralism* (Routledge 2008) 97. On the concept of *différance*, see Derrida, *Marges de la Philosophie* (n 42) 1–29; Derrida, *Positions* (n 14) 17 and 37–41. See the application of the concept of *différance* by Jacques Derrida, *The Beast and the Sovereign*, vol 1 (University of Chicago Press 2011). See the comments of Bennington (n 41) 70–82; Salmon (n 41) 107. See also Serpil Tunç Utebay, *Justice en tant que loi, justice au-delà de la loi. Hobbes, Derrida et les Critical Legal Studies* (L’Harmattan 2017) 79–81. On the kinship between Derrida’s *différance* and the work of Heidegger, see Walter A Brogan, ‘The Original Difference’ in David Wood and Robert Bernasconi (eds), *Derrida and Différance* (Northwestern UP 1988) 31.

43 This is the presupposition of what has been called the transcendental signified. See Derrida, *De la Grammatologie* (n 30) 24 and 69–70; Derrida, *Positions* (n 14) 30 ; Merleau-Ponty (n 23) 70–3. For some illustrations of the work of self-difference in relation to legal texts, see Forray and Pimont (n 33) 219–22.

44 On the notion of trace, see generally Derrida, *De la Grammatologie* (n 30) 86–7; Bennington (n 41) 73–5. See also the remarks of Utebay (n 42) 81–2. On the idea that trace haunts the text, see generally Jacques Derrida, *Spectres de Marx* (Galilée 1993). See also Jacques Derrida, *Papier Machine* (n 20) 307. On the question of spectral dimension of legal texts, see Legrand, ‘Siting Foreign Law’ (n 22) 607; Pierre Legrand, ‘Foreign Law: Understanding Understanding’ (2011) 6 Journal of Comparative Law 67, 78.

45 Legrand, ‘Foreign Law: Understanding Understanding’ (n 44) 82.

46 Pierre Legrand, ‘“Il n’y a pas de hors-texte:” Intimations of Jacques Derrida as Comparatist-at-Law’ in Peter Goodrich and others (eds), *Derrida and Legal Philosophy* (Palgrave MacMillan 2008) 131; see also Legrand, ‘Siting Foreign Law’ (n 22) 607.

47 Derrida claims that this self-difference is out of both the order of representation and the order of things, and approaches ‘it’ as neither a form nor a thing. Self-difference is out of space, temporality, causality and sensibility, as well as out of representation. See Derrida, *Marges de la Philosophie* (n 42) 1–29. On the idea that the trace refers to the remnants of what is left, see Derrida, *Papier Machine* (n 20) 385. With a specific emphasis on legal studies, see the remarks of Legrand, ‘Foreign Law: Understanding Understanding’ (n 44) 79.

48 On the idea of ghost, see Legrand, ‘Siting Foreign Law’ (n 22) 607.

49 Legrand, ‘Foreign Law: Understanding Understanding’ (n 44) 79; Legrand, ‘“Il n’y a pas de hors-texte”’ (n 46) 131.

50 Legrand, ‘Foreign Law: Understanding Understanding’ (n 44) 82.

51 In the same vein and in relation to legal studies, see JM Balkin, ‘Deconstructive Practice and Legal Theory’ (1987) 96 Yale LJ 743.

52 Legrand, ‘Foreign Law: Understanding Understanding’ (n 44) 79.

53 Derrida, *Positions* (n 14) 39. See also Zarbiyev (n 11) 43–5. For a discussion of this idea in relation to legal studies, see Legrand, ‘Foreign Law: Understanding Understanding’ (n 44) 82; Legrand, ‘Siting Foreign Law’ (n 22) 609.

54 Derrida himself was rather ambiguous on this point. On the one hand, he claims that the work of self-identity is not binary (see Derrida, *Positions* (n 14) 39; Malabou and Derrida (n 20) 73). On the other hand, in a move that he tries to strip of its Hegelian overtones, he claims that deconstruction must reverse hierarchies (see Derrida, *Positions* (n 14) 56–61). On this latter aspect of Derrida, see Salmon (n 41) 81.

55 Reducing the divided identity of forms to a binary identity has often been witnessed in critical legal scholarship. See eg Balkin, ‘Deconstructive Practice and Legal Theory’ (n 51) 754. This is yet another reason why critical legal scholarship has remained very meaning-centric.

56 Pierre Legrand writes that if interpreters seek to determine meaning, they are condemned to impotence: Legrand, ‘Foreign Law: Understanding Understanding’ (n 44) 84.

57 Derrida, *Marges de la Philosophie* (n 42) 17; Barthes, *Le bruissement de la langue* (n 41) 47. On the earlier idea that the language is not at the service of signification but is signification itself, see Merleau-Ponty (n 23) 68–9 and 379–80.

58 Legrand, ‘“Il n’y a pas de hors-texte”’ (n 46) 131.

59 See Roland Barthes, *L’aventure sémiologique* (Seuil 1985) 13; Barthes, *Le bruissement de la langue* (n 41) 56 ; Barthes, *S/Z* (n 41) 10–11; Barthes, *Critique et vérité* (n 15) 59.

60 On the idea of text as an event, see Pierre Legrand, ‘The Same and the Different’ in Pierre Legrand and Roderick Munday (eds), *Comparative Legal Studies: Traditions and Transitions* (CUP 2003) 244. See also Legrand, ‘“Il n’y a pas de hors-texte”’ (n 46) 143.

61 On the idea of passage in the text, see Barthes, *Le bruissement de la langue* (n 41) 75. On the idea that the text is a volume of traces and deferrals, see Barthes, *L’aventure sémiologique* (n 59) 13.

62 On the idea of wandering, see Goodrich (n 19) 2063. Pierre Legrand speaks of tracing as meandering. See Legrand, ‘Foreign Law: Understanding Understanding’ (n 44) 154.

63 Barthes, *S/Z* (n 41) 17.

64 Derrida, *De la Grammatologie* (n 30) 125.

65 Goodrich (n 19) 2048. Appreciating the text’s plenitude is also part of Edward Morgan’s ambitious comparison of the literary and aesthetic moves in international legal literature with those observed in modern literature. See Morgan (n 18).

66 Barthes, *S/Z* (n 41) 11 and 20. In the same vein, see Legrand, ‘“Il n’y a pas de hors-texte”’ (n 46) 131.

67 Barthes, *Le bruissement de la langue* (n 41) 66.

68 Jacques Derrida, *La Carte Postale* (Flammarion 1980) 10 (‘il est mauvais de prédestiner sa lecture’).

69 For a similar understanding of Derrida’s work, see Culler, *On Deconstruction* (n 42) ii. See also Peter Goodrich and others, ‘Introduction: A Philosophy of Legal Enigmas’ in Goodrich and others (n 46) 12. See also Simon Critchley, ‘Derrida’s Influence on Philosophy… And on My Work’ (2005) 6 German Law Journal 25, 27; Anne Orford, ‘Critical Intimacies: Reading International Law’ in Goodrich and others (n 46) 115; Anne Orford, ‘Critical Intimacy: Jacques Derrida and the Friendship of Politics’ (2005) 6 German Law Journal 31, 31.

70 Derrida, *La Carte Postale* (n 68) 11.

71 Avita Ronell, ‘Saying Goodbye: An Amateur Video’ in Goodrich and others (n 46) 245.

72 On the idea of the science of reading as a science of inexhaustion, see Barthes, *Le bruissement de la langue* (n 41) 47. On the idea that interpretation of the legal text can never be completed, see Legrand, ‘Foreign Law: Understanding Understanding’ (n 44) 84. In the same vein, see Tzouvala (n 11) 14.

73 On the idea of over-interpretation, see Derrida, *Papier Machine* (n 20) 27. In relation to international legal studies, see Tzouvala (n 11) 13.

74 Deleuze and Parnet (n 8) 21 and 27.

75 On the idea that volume is intolerable to traditional dualism, see Derrida, *L’écriture et la différence* (n 6) 42.

76 For a promotion of oppositional reading in international legal studies, see Tzouvala (n 11) 9–10 (drawing on I Bennett Capers, ‘Reading Back, Reading Black’ (2006) 35 Hofstra L Rev 9). See also Juan M Amaya-Castro and Hassan El Menyawi, ‘Moving Away from Moving Away: A Conversation about Jacques Derrida and Legal Scholarship’ (2005) 6 German Law Journal 101, 109–10. For the promotion of oppositional reading in legal studies in general, see Balkin, ‘Deconstructive Practice and Legal Theory’ (n 51) 765; JM Balkin, ‘Deconstruction’s Legal Career’ (2005) 27 Cardozo L Rev 719, 722–9.

77 See s 4A above.

78 For some similar reservations towards the methodological unpicking of binary oppositions, see Critchley (n 69) 26.

79 On the idea that the form, if allocated a definite and fixed meaning, ceases to be a form, see Barthes, *Le bruissement de la langue* (n 41) 109.

80 On the idea that the more literary theory is resisted the more it flourishes, see Paul de Man, *The Resistance to Theory* (University of Minnesota Press 1986) 19–20.

81 In the same vein, J Derrida, ‘Force of Law—The ‘Mystical Foundation of Authority’’ in Gil Anidjar (ed), *Acts of Religion—Jacques Derrida* (Routledge) 242–3.

82 David Kennedy, ‘Critical Theory, Structuralism and Contemporary Legal Scholarship’ (1985–86) 21 New Eng L Rev 209, 287. See also the remarks of Jean d’Aspremont, ‘A Worldly Law in a Legal World’ in Andrea Bianchi and Moshe Hirsch (eds), *International Law’s Invisible Frames* (OUP 2021).

83 The metaphor is from Deleuze and Parnet (n 8) 40. On the idea of the meaning being nomadic in legal texts, see Legrand, ‘“Il n’y a pas de hors-texte”’ (n 46) 131.

84 Against the idea that the removal of the signified entails a removal of human control, see Harland (n 40) 135.

85 Pierre Legrand, ‘On the Singularity of Law’ (2006) 47 Harv Int’l LJ 517, 526.

86 See Jacques Derrida, ‘The Force of Law: The “Mystical Foundation of Authority”’ (1989–90) 11 Cardozo L Rev 920, 956 and 961. See also Derrida, *Papier Machine* (n 20) 341–2; Goodrich (n 19) 2068; Legrand, ‘Siting Foreign Law’ (n 22) 624; Forray and Pimont (n 33) 343–66; Petra Gehring, ‘Force and “Mystical Foundation” of Law: How Jacques Derrida Addresses Legal Discourse’ (2005) 6 German Law Journal 151; Adam Thurschwell, ‘Specters and Scholars: Derrida and the Tragedy of Political Thought’ (2005) 6 German Law Journal 87; Florian Hoffmann, ‘Epilogue: In Lieu of conclusion’ (2005) 6 German Law Journal 197, 199; Costas Douzinas, ‘Violence, Justice, Deconstruction’ (2005) 6 German Law Journal 171.

87 This is a point that I owe to one of the anonymous reviewers.

88 This is a criticism raised by Régis Debray against literary theory. See Régis Debray, *Cours de médiologie générale* (Gallimard 2001) 75.

89 This is the criticism of Quentin Skinner against literary theory. See Quentin Skinner, *The Foundations of Modern Political Thought*, vol 1 (CUP 1998) xi and xiii.

90 This is a criticism of Edward Said against post-structuralist literary theory. See Said (n 10) 4.

91 See generally Ingo Venzke and Kevin Jon Heller, *Contingency in International Law. On the Possibility of Different Legal Histories* (OUP 2021).

92 On the turn to history in contemporary international legal scholarship, see Matthew Craven, ‘Theorizing the Turn to History in International Law’ in Anne Orford and Florian Hoffmann (eds), *The Oxford Handbook of the Theory of International Law* (O UP 2016) 21–37; see also George Galindo, ‘Martti Koskenniemi and the Historiographical Turn in International Law’ (2005) 16 EJIL 539.

93 See eg Monica Hakimi, ‘The Work of International Law’ (2017) 58 Harv Int’l LJ 1; David Kennedy, *A World of Struggle: How Power, Law, and Expertise Shape Global Political Economy* (Princeton UP 2016); M Koskenniemi, ‘The Politics of International Law—20 Years Later’ (2009) 20 EJIL 7; Venzke (n 26); Sundhya Pahuja, ‘Decolonization and the Eventness of International Law’ in Fleur Johns, Richard Joyce and Sundhya Pahuja (eds), *Events: The Force of International Law* (Routledge 2011) 91–105.

94 See the remarks of Jürgen Habermas in Giovanna Borradori, *Philosophy in a Time of Terror. Dialogues with Jürgen Habermas and Jacques Derrida* (University of Chicago Press 2003) 13–15. See also Jürgen Habermas, *The Philosophical Discourse of Modernity. Twelve Lectures* (Frederik Lawrence tr, Polity Press 1987) 161–210.

95 This is a criticism made by Rorty against post-structuralist literary theory. See Richard Rorty, *Consequences of Pragmatism* (University of Minneapolis Press 2011) 150–1. For similar criticisms in the international legal literature, see generally Carty (n 36).

96 Derrida, *L’écriture et la différence* (n 6) 427; Culler, *On Deconstruction* (n 42) 132. See also the remarks of Balkin, ‘Deconstructive Practice and Legal Theory’ (n 51) 776–7 and 785; Balkin, ‘Deconstruction’s Legal Career’ (n 76) 719.

97 This is a charge made by Culler, *Structuralist Poetics* (n 6) 289.

98 ibid 295.

99 On the idea of naturalistic necessity, see Judith Butler, *Gender Trouble* (2nd edn, Routledge 2007) 45.

